# Supplementary material for: Development, and Internal, and External Validation of a Scoring System to Predict 30-Day Mortality after Having a Traffic Accident Traveling by Private Car or Van: An Analysis of 164,790 Subjects and 79,664 Accidents
Source: Int J Environ Res Public Health. 2020 Dec 18;17(24):9518. doi: 10.3390/ijerph17249518 (PMC7766065; doi:10.3390/ijerph17249518)
Supplement: Supplementary file 1 [file ijerph-17-09518-s001.zip › Table S1.pdf]

Table S1: Spline functions for the age variable.

Age (years)	S1	S2	S3	S4	S5	S6
**0**	0.00	0.00	0.00	0.00	0.00	0.00
**1**	0.11	0.00	0.00	0.00	0.00	0.00
**2**	0.21	0.01	0.00	0.00	0.00	0.00
**3**	0.30	0.02	0.00	0.00	0.00	0.00
**4**	0.37	0.03	0.00	0.00	0.00	0.00
**5**	0.43	0.05	0.00	0.00	0.00	0.00
**6**	0.48	0.07	0.00	0.00	0.00	0.00
**7**	0.53	0.10	0.00	0.00	0.00	0.00
**8**	0.56	0.12	0.01	0.00	0.00	0.00
**9**	0.58	0.15	0.01	0.00	0.00	0.00
**10**	0.59	0.18	0.01	0.00	0.00	0.00
**11**	0.60	0.21	0.01	0.00	0.00	0.00
**12**	0.60	0.24	0.02	0.00	0.00	0.00
**13**	0.59	0.28	0.02	0.00	0.00	0.00
**14**	0.58	0.31	0.03	0.00	0.00	0.00
**15**	0.56	0.34	0.04	0.00	0.00	0.00
**16**	0.54	0.37	0.04	0.00	0.00	0.00
**17**	0.51	0.41	0.05	0.00	0.00	0.00
**18**	0.48	0.44	0.06	0.00	0.00	0.00
**19**	0.45	0.46	0.07	0.00	0.00	0.00
**20**	0.42	0.49	0.09	0.00	0.00	0.00
**21**	0.38	0.52	0.10	0.00	0.00	0.00

**22**	0.35	0.54	0.11	0.00	0.00	0.00
**23**	0.31	0.56	0.13	0.00	0.00	0.00
**24**	0.28	0.57	0.15	0.00	0.00	0.00
**25**	0.25	0.58	0.17	0.00	0.00	0.00
**26**	0.22	0.59	0.19	0.00	0.00	0.00
**27**	0.19	0.60	0.21	0.00	0.00	0.00
**28**	0.17	0.60	0.23	0.00	0.00	0.00
**29**	0.15	0.59	0.26	0.00	0.00	0.00
**30**	0.13	0.59	0.28	0.00	0.00	0.00
**31**	0.11	0.58	0.31	0.00	0.00	0.00
**32**	0.09	0.57	0.33	0.00	0.00	0.00
**33**	0.08	0.55	0.36	0.01	0.00	0.00
**34**	0.07	0.54	0.39	0.01	0.00	0.00
**35**	0.05	0.52	0.41	0.01	0.00	0.00
**36**	0.04	0.50	0.44	0.01	0.00	0.00
**37**	0.04	0.48	0.47	0.02	0.00	0.00
**38**	0.03	0.46	0.49	0.02	0.00	0.00
**39**	0.02	0.43	0.51	0.03	0.00	0.00
**40**	0.02	0.41	0.54	0.04	0.00	0.00
**41**	0.01	0.38	0.56	0.04	0.00	0.00
**42**	0.01	0.36	0.58	0.05	0.00	0.00
**43**	0.01	0.33	0.60	0.06	0.00	0.00
**44**	0.00	0.31	0.61	0.08	0.00	0.00
**45**	0.00	0.28	0.63	0.09	0.00	0.00

**46**	0.00	0.26	0.64	0.10	0.00	0.00
**47**	0.00	0.23	0.65	0.12	0.00	0.00
**48**	0.00	0.21	0.66	0.13	0.00	0.00
**49**	0.00	0.19	0.66	0.15	0.00	0.00
**50**	0.00	0.17	0.66	0.17	0.00	0.00
**51**	0.00	0.15	0.66	0.19	0.00	0.00
**52**	0.00	0.13	0.65	0.22	0.00	0.00
**53**	0.00	0.11	0.65	0.24	0.00	0.00
**54**	0.00	0.10	0.64	0.26	0.00	0.00
**55**	0.00	0.09	0.62	0.29	0.00	0.00
**56**	0.00	0.07	0.61	0.32	0.00	0.00
**57**	0.00	0.06	0.59	0.34	0.01	0.00
**58**	0.00	0.05	0.57	0.37	0.01	0.00
**59**	0.00	0.04	0.55	0.39	0.01	0.00
**60**	0.00	0.04	0.53	0.42	0.02	0.00
**61**	0.00	0.03	0.50	0.44	0.02	0.00
**62**	0.00	0.02	0.48	0.47	0.03	0.00
**63**	0.00	0.02	0.45	0.49	0.04	0.00
**64**	0.00	0.01	0.43	0.51	0.05	0.00
**65**	0.00	0.01	0.40	0.53	0.06	0.00
**66**	0.00	0.01	0.37	0.55	0.07	0.00
**67**	0.00	0.01	0.35	0.56	0.09	0.00
**68**	0.00	0.00	0.32	0.58	0.10	0.00
**69**	0.00	0.00	0.29	0.59	0.12	0.00

**70**	0.00	0.00	0.27	0.59	0.14	0.00
**71**	0.00	0.00	0.24	0.60	0.16	0.00
**72**	0.00	0.00	0.22	0.60	0.18	0.00
**73**	0.00	0.00	0.19	0.60	0.21	0.00
**74**	0.00	0.00	0.17	0.59	0.24	0.00
**75**	0.00	0.00	0.15	0.58	0.27	0.00
**76**	0.00	0.00	0.13	0.56	0.30	0.00
**77**	0.00	0.00	0.11	0.54	0.34	0.00
**78**	0.00	0.00	0.10	0.52	0.38	0.00
**79**	0.00	0.00	0.09	0.50	0.41	0.01
**80**	0.00	0.00	0.07	0.47	0.45	0.01
**81**	0.00	0.00	0.06	0.44	0.48	0.02
**82**	0.00	0.00	0.05	0.41	0.51	0.03
**83**	0.00	0.00	0.04	0.37	0.54	0.04
**84**	0.00	0.00	0.03	0.34	0.57	0.06
**85**	0.00	0.00	0.03	0.30	0.59	0.08
**86**	0.00	0.00	0.02	0.27	0.60	0.11
**87**	0.00	0.00	0.02	0.23	0.61	0.14
**88**	0.00	0.00	0.01	0.20	0.61	0.18
**89**	0.00	0.00	0.01	0.17	0.60	0.23
**90**	0.00	0.00	0.01	0.14	0.58	0.28
**91**	0.00	0.00	0.00	0.11	0.55	0.34
**92**	0.00	0.00	0.00	0.08	0.51	0.40
**93**	0.00	0.00	0.00	0.06	0.46	0.48

**94**	0.00	0.00	0.00	0.04	0.40	0.56
**95**	0.00	0.00	0.00	0.02	0.32	0.66
**96**	0.00	0.00	0.00	0.01	0.23	0.76
**98**	0.00	0.00	0.00	0.00	0.00	1.00

Abbreviations: S, B-spline function.
