# Supplementary material for: Development, and Internal, and External Validation of a Scoring System to Predict 30-Day Mortality after Having a Traffic Accident Traveling by Private Car or Van: An Analysis of 164,790 Subjects and 79,664 Accidents
Source: Int J Environ Res Public Health. 2020 Dec 18;17(24):9518. doi: 10.3390/ijerph17249518 (PMC7766065; doi:10.3390/ijerph17249518)
Supplement: Supplementary file 1 [file ijerph-17-09518-s001.zip › Table S2.pdf]

Table S2: Spline functions for the time variable.

Time (hours)	S1	S2	S3	S4	S5	S6
**0**	0.00	0.00	0.00	0.00	0.00	0.00
**1**	0.38	0.04	0.00	0.00	0.00	0.00
**2**	0.56	0.13	0.01	0.00	0.00	0.00
**3**	0.59	0.26	0.02	0.00	0.00	0.00
**4**	0.52	0.40	0.05	0.00	0.00	0.00
**5**	0.39	0.51	0.10	0.00	0.00	0.00
**6**	0.25	0.58	0.17	0.00	0.00	0.00
**7**	0.14	0.59	0.26	0.00	0.00	0.00
**8**	0.07	0.55	0.37	0.01	0.00	0.00
**9**	0.03	0.47	0.48	0.02	0.00	0.00
**10**	0.01	0.37	0.57	0.05	0.00	0.00
**11**	0.00	0.26	0.64	0.10	0.00	0.00
**12**	0.00	0.17	0.66	0.18	0.00	0.00
**13**	0.00	0.10	0.63	0.28	0.00	0.00
**14**	0.00	0.05	0.55	0.39	0.01	0.00
**15**	0.00	0.02	0.45	0.49	0.04	0.00
**16**	0.00	0.01	0.34	0.57	0.09	0.00
**17**	0.00	0.00	0.23	0.60	0.17	0.00
**18**	0.00	0.00	0.13	0.57	0.30	0.00
**19**	0.00	0.00	0.07	0.47	0.46	0.01
**20**	0.00	0.00	0.03	0.32	0.59	0.06
**21**	0.00	0.00	0.01	0.17	0.61	0.22

**22**	0.00	0.00	0.00	0.05	0.44	0.51
**23**	0.00	0.00	0.00	0.00	0.00	1.00

Abbreviations: S, B-spline function.
