# Supplementary material for: Development, and Internal, and External Validation of a Scoring System to Predict 30-Day Mortality after Having a Traffic Accident Traveling by Private Car or Van: An Analysis of 164,790 Subjects and 79,664 Accidents
Source: Int J Environ Res Public Health. 2020 Dec 18;17(24):9518. doi: 10.3390/ijerph17249518 (PMC7766065; doi:10.3390/ijerph17249518)
Supplement: Supplementary file 1 [file ijerph-17-09518-s001.zip › Table S3.pdf]

Table S3: Spline functions for the age of the vehicle variable.

Age (years)	S1	S2	S3	S4	S5	S6
**0**	0.00	0.00	0.00	0.00	0.00	0.00
**1**	0.43	0.05	0.00	0.00	0.00	0.00
**2**	0.59	0.18	0.01	0.00	0.00	0.00
**3**	0.56	0.34	0.04	0.00	0.00	0.00
**4**	0.42	0.49	0.09	0.00	0.00	0.00
**5**	0.25	0.58	0.17	0.00	0.00	0.00
**6**	0.13	0.59	0.28	0.00	0.00	0.00
**7**	0.05	0.52	0.42	0.00	0.00	0.00
**8**	0.02	0.41	0.57	0.00	0.00	0.00
**9**	0.00	0.28	0.70	0.01	0.00	0.00
**10**	0.00	0.17	0.81	0.02	0.00	0.00
**11**	0.00	0.09	0.88	0.04	0.00	0.00
**12**	0.00	0.04	0.90	0.06	0.00	0.00
**13**	0.00	0.01	0.91	0.08	0.00	0.00
**14**	0.00	0.00	0.89	0.11	0.00	0.00
**15**	0.00	0.00	0.86	0.13	0.00	0.00
**16**	0.00	0.00	0.84	0.16	0.00	0.00
**17**	0.00	0.00	0.81	0.18	0.01	0.00
**18**	0.00	0.00	0.79	0.20	0.01	0.00
**19**	0.00	0.00	0.76	0.22	0.01	0.00
**20**	0.00	0.00	0.74	0.24	0.02	0.00
**21**	0.00	0.00	0.72	0.26	0.02	0.00

**22**	0.00	0.00	0.69	0.28	0.02	0.00
**23**	0.00	0.00	0.67	0.30	0.03	0.00
**24**	0.00	0.00	0.65	0.31	0.04	0.00
**25**	0.00	0.00	0.63	0.33	0.04	0.00
**26**	0.00	0.00	0.61	0.34	0.05	0.00
**27**	0.00	0.00	0.59	0.36	0.05	0.00
**28**	0.00	0.00	0.57	0.37	0.06	0.00
**29**	0.00	0.00	0.55	0.38	0.07	0.00
**30**	0.00	0.00	0.53	0.39	0.08	0.00
**31**	0.00	0.00	0.51	0.40	0.08	0.00
**32**	0.00	0.00	0.49	0.41	0.09	0.01
**33**	0.00	0.00	0.47	0.42	0.10	0.01
**34**	0.00	0.00	0.46	0.43	0.11	0.01
**35**	0.00	0.00	0.44	0.43	0.12	0.01
**36**	0.00	0.00	0.42	0.44	0.13	0.01
**37**	0.00	0.00	0.41	0.45	0.14	0.01
**38**	0.00	0.00	0.39	0.45	0.15	0.01
**39**	0.00	0.00	0.38	0.46	0.15	0.01
**40**	0.00	0.00	0.36	0.46	0.16	0.02
**41**	0.00	0.00	0.35	0.46	0.17	0.02
**42**	0.00	0.00	0.33	0.46	0.18	0.02
**43**	0.00	0.00	0.32	0.46	0.19	0.02
**44**	0.00	0.00	0.31	0.47	0.20	0.03
**45**	0.00	0.00	0.29	0.47	0.21	0.03

**46**	0.00	0.00	0.28	0.47	0.22	0.03
**47**	0.00	0.00	0.27	0.46	0.23	0.03
**49**	0.00	0.00	0.24	0.46	0.25	0.04
**50**	0.00	0.00	0.23	0.46	0.26	0.04
**51**	0.00	0.00	0.22	0.45	0.27	0.05
**67**	0.00	0.00	0.09	0.35	0.41	0.14
**77**	0.00	0.00	0.05	0.26	0.45	0.25
**103**	0.00	0.00	0.00	0.03	0.26	0.70
**114**	0.00	0.00	0.00	0.00	0.00	1.00

Abbreviations: S, B-spline function.
