# Supplementary figures and images for: Development, and Internal, and External Validation of a Scoring System to Predict 30-Day Mortality after Having a Traffic Accident Traveling by Private Car or Van: An Analysis of 164,790 Subjects and 79,664 Accidents
Source: Int J Environ Res Public Health. 2020 Dec 18;17(24):9518. doi: 10.3390/ijerph17249518 (PMC7766065; doi:10.3390/ijerph17249518)

## 30-day mortality risk

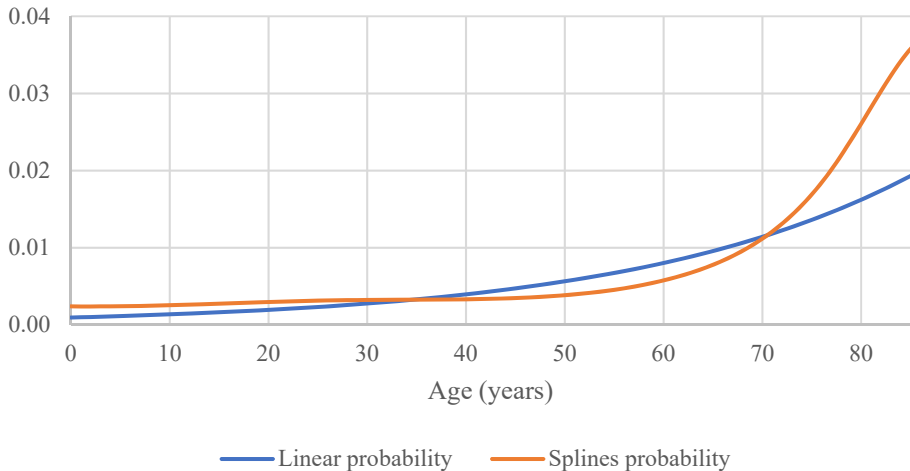

Supplement: Supplementary file 1 [file ijerph-17-09518-s001.zip › Figure S1.pdf]

## 30-day mortality risk

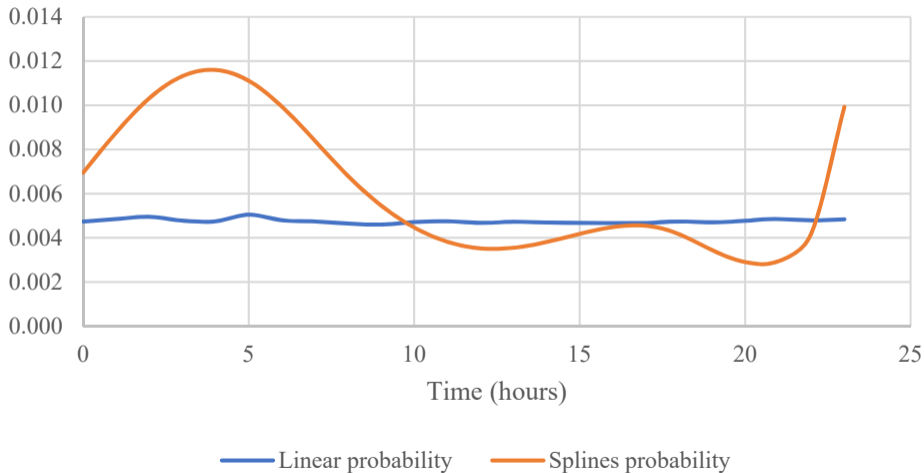

Supplement: Supplementary file 1 [file ijerph-17-09518-s001.zip › Figure S2.pdf]

## 30-day mortality risk

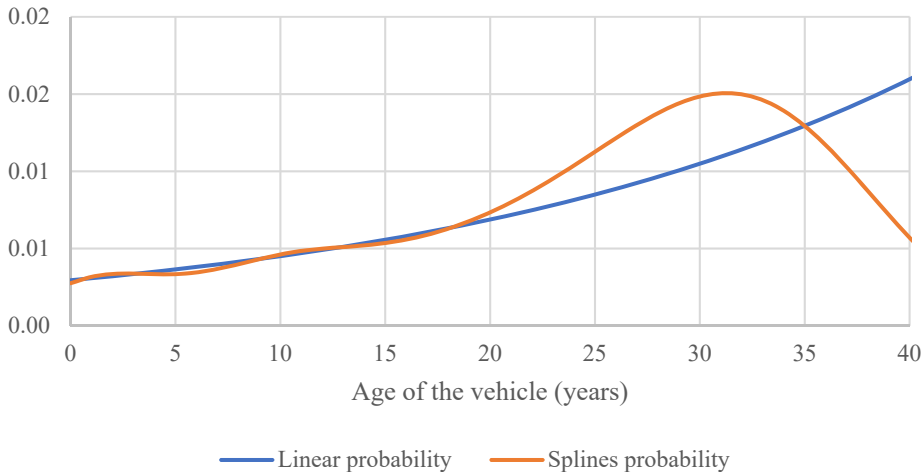

Supplement: Supplementary file 1 [file ijerph-17-09518-s001.zip › Figure S3.pdf]
